# Whole genome sequence of *Penicillium kloeckeri* and insight into its growth-promoting, saline alkaline tolerance properties

**DOI:** 10.3389/fmicb.2025.1675497

**Published:** 2025-10-10

**Authors:** Wenxiao Cui, Yafen Wu, Bin Ni, Jia Cao

**Affiliations:** ^1^College of Resources and Environmental Sciences, China Agricultural University, Beijing, China; ^2^Beijing Key Laboratory of Biodiversity and Organic Farming, China Agricultural University, Beijing, China; ^3^State Key Laboratory of Nutrient Use and Management, College of Resources and Environmental Sciences, China Agricultural University, Beijing, China

**Keywords:** *Penicillium kloeckeri*, whole-genome sequencing, untargeted metabolomics, soybean hydroponic experiment, saline alkali soil

## Abstract

**Introduction:**

Microbial agents, mainly bacterial strains, are widely used in agriculture, their often limited efficacy underscores the critical need for resilient fungal alternatives. A novel strain of *Penicillium kloeckeri* with potential agricultural applications was isolated and purified.

**Methods and Results:**

Here, the genome of *P. kloeckeri* was sequenced and function predicted. The assembled *P. kloeckeri* genome had a 34.28 Mb genome size with 41.87% GC content, and revealed functional genes associated with Indole-3-Acetic Acid (IAA), daidzein, siderophore, and organic acid secretion, suggesting its role in plant growth promotion and soil improvement. Metabolite analysis further confirmed daidzein and amino acid production. Under salt stress conditions, *P. kloeckeri* inoculation (YJKS) significantly improved soybean growth compared to the salt-stressed control (YCK). Specifically, inoculation increased stem diameter by 17.79% and dramatically enhanced root length (99.95%), root area (111.96%), root volume (114.80%), and root biomass (14.97%). Furthermore, in saline alkali soil, *P. kloeckeri* inoculation effectively reduced soil pH, improve soil physical structure, and enhance nutrient availability.

**Discussion:**

*P. kloeckeri* is a promising new fungal strain with potential agricultural applications. This study provides a foundational genomic resource and initial evidence for its agricultural utility, warranting further investigation into its underlying mechanisms and commercial viability.

## 1 Introduction

Soil microorganisms represent one of the most diverse biological communities on Earth. A single gram of soil can contain billions of microbial cells and thousands of distinct species (Ma et al., 2023). These communities include bacteria, fungi, actinomycetes, and algae, which interact with one another and with plants to form complex ecological networks (Zhang et al., 2021; Ruiu, 2020; Shen et al., 2024). Over 450 million years of co-evolution have shaped the relationship between plants and microorganisms, giving microbes a central role in ecosystem functioning. (Cordovez et al., 2019; Hassani et al., 2018; Coban et al., 2022). Within agricultural systems, microorganisms support plant growth by supplying nutrients, improving the rhizosphere environment, and enhancing resistance to environmental stresses (Ku et al., 2024; Shi et al., 2024; Zheng et al., 2024). Consequently, they are considered indispensable for soil health, crop productivity, and agricultural sustainability (Gao et al., 2024). In current agricultural practice, most commercial microbial products are based on bacteria. Plant Growth-Promoting Rhizobacteria (PGPR) have been widely tested for their ability to stimulate plant growth and improve soil fertility. For example, nitrogen-fixing bacteria were used to increase nitrogen use efficiency (Chen et al., 2024) while phosphorus-solubilizing bacteria enhanced the availability of insoluble phosphorus sources (Chang et al., 2024). However, fungi, despite their ecological importance and functional versatility, remain underrepresented in microbial inoculants (Stukenbrock and Gurr, 2023). Research shows that fungi act as decomposers, symbionts, and pathogens in natural ecosystems (Yang et al., 2017), and they also contribute to soil structure improvement, water-use efficiency, and plant stress resistance in agricultural soils (Li et al., 2020). Mycorrhizal fungi, for instance, increase nutrient uptake and enhance plant tolerance to drought, salinity, and pests (Wang et al., 2024). Compared with bacteria, fungi may provide additional benefits due to their unique cellular structures and greater tolerance to environmental stresses (Wang and Kuzyakov, 2024). Nevertheless, their practical application is still limited, as fungal strains accounted for only 14.4% of newly registered microbial fertilizers between January and June 2024, whereas Bacillus-based products dominated the market (Sánchez et al., 2024). These observations highlight the substantial but underexploited potential of fungi in agriculture and underscore the need for further research and development.

Fungi are key players in natural ecosystems because of their unique biological traits and extensive biomass in soil (Coleine et al., 2022; Jiao et al., 2022). Existing data indicate that in the top 30 cm of soil, the total length of fungal hyphae per cubic centimeter of soil averages 1,020 meters (Sokol et al., 2022). Their hyphal networks not only establish symbiotic associations with plants but also contribute substantially to nutrient cycling. As decomposers, fungi convert complex organic matter into accessible nutrients, while as symbionts or pathogens they engage in intricate interactions with plants, animals, and other microorganisms, thereby maintaining ecosystem stability (Wu et al., 2024; Genre et al., 2020). Within this group, *Penicillium* is particularly important due to its species richness, global distribution, and strong secondary metabolic capacity. These fungi secrete organic acids and enzymes that dissolve insoluble phosphorus and potassium, regulate soil pH, and improve soil fertility (Jo et al., 2023; Zhang et al., 2024). In addition, their hyphal growth and exudates promote soil aggregate formation, enhance soil structure, and improve nutrient availability (Wu et al., 2023). *Penicillium* also exhibits high cellulase activity, supporting carbon turnover and nutrient cycling (Jiang et al., 2020). Beyond soil fertility, fungi contribute to plant resilience under stress conditions. Their metabolites can stimulate lignification in plant cell walls, induce stress-related compounds, and improve tolerance to biological stress, drought, salinity, and temperature extremes (Sánchez et al., 2024). For example, *Penicillium citrinum* YW322 suppressed ginseng root rot by inhibiting *Aspergillus* spp. and modulating rhizosphere microbial communities (Wang et al., 2022). Similarly, Paria et al. (2022) utilized the ability of *Aspergillus penicillioides* (F12) to produce large amounts of exopolysaccharides (EPS). This ability allowed the bioremediation of heavy metals, such as lead, in wastewater. The process achieved a remarkable adsorption rate of 73.14%. These findings demonstrate that fungi hold considerable promise for application in challenging environments such as saline-alkaline or acidic soils. However, most studies remain descriptive, and the underlying molecular mechanisms of fungal-mediated growth promotion and stress tolerance are still poorly understood (Rana et al., 2024). In order to further reveal this mechanism and process, changes and integration in the research methods are necessary.

WGS has been widely applied to improve species identification, predict functional genes, and reconstruct metabolic pathways (Liu et al., 2022; Ramsamy et al., 2020; Kweon et al., 2020). These applications provided valuable insights into microbial functions such as organic matter degradation, nitrogen fixation, and stress resistance, thereby highlighting the essential ecological roles of microorganisms in soil and plants (Chetverikov et al., 2023; Merker et al., 2020; Fan et al., 2024; Zhu et al., 2024). However, WGS alone is insufficient to fully resolve questions related to metabolic adaptability and regulatory mechanisms under environmental stress (Nagy et al., 2023). To complement genomic data, researchers have used metabolomics to capture dynamic changes in small-molecule metabolites. This approach linked gene expression to phenotypic traits and extended the mechanistic understanding gained from genome sequencing (Cui et al., 2024; Ferrell et al., 2024). For instance, the endophytic bacteria were used to reveal the molecular mechanism of alleviating the nutritional limitation of rapeseed growth under low phosphorus environment by multi-omics combined methods (Liu et al., 2024). These findings suggest that integrating WGS with metabolomics and other omics approaches is essential for dissecting the complex regulatory mechanisms of fungi. Such combined strategies not only bridge the gap between genotype and phenotype but also lay a robust foundation for the development of fungal inoculants with improved agricultural applications.

In this study, a strain of *P. kloeckeri* with potential agricultural applications is investigated through an integrated approach that combines WGS, metabolomic profiling, and phenotypic validation. WGS provides the complete genomic sequence, enabling the identification of functional genes, regulatory elements, and agriculturally relevant pathways. Metabolomic analysis uncovers metabolic responses under salt stress, while phenotypic experiments confirm the effects of this strain on soybean growth promotion and salt tolerance. By linking genomic potential with metabolic activity and observable phenotypes, this study offers new insights into the molecular mechanisms underlying fungal-mediated plant growth promotion and soil improvement.

## 2 Materials and methods

### 2.1 Fungal strains and culture conditions

The fungal strain was isolated from the rhizosphere of soybean plants in the soybean-growing region of Heilongjiang Province, China. Isolation and purification of the strain was performed in the Soil Ecology Laboratory of China Agricultural University. The strain was cultured on Potato Dextrose Agar (PDA) plates at 25 °C for 7 days. Conidia from the 7 day old PDA cultures were harvested by washing the plates with sterile distilled water.

### 2.2 Genome sequencing and assembly

Conidia of the strain were inoculated into sterilized PDA liquid medium and incubated at 25 °C with shaking at 200 r/min for 48 h. The resulting mycelia were collected by filtration, washed three times with sterile water, and subsequently frozen and ground in liquid nitrogen. Genomic DNA was extracted using the Ezup Column Fungal DNA Extraction Kit (Sangon Biotech, Shanghai, China) and further purified with the Genomic DNA Purification Kit (Thermo Fisher Scientific, USA) according to the manufacturer's instructions. Genome sequencing was carried out in two stages using Illumina and PacBio technologies. Illumina sequencing was performed on a HiSeq2000 instrument using two genomic libraries: 180 bp (paired-end) and 2 Kb (mate-pair) insert sizes, with 100 bp read length. Second, PacBio sequencing technology (model RSII and P4C2 chemistry) was used to produce long reads (>1 Kb).

Genome assembly was performed in two steps. Firstly, Illumina reads were assembled with ALLPATHS-LG (version r43019) (Genre et al., 2020) at 200 × coverage to generate high-quality contigs and scaffolds. Secondly, scaffolding was improved using PacBio long reads with SSPACE-LongRead (version 1.1). Assembly quality was assessed using using CEGMA (Paria et al., 2022) by aligning 248 highly conserved eukaryotic proteins to estimate genome completeness. Additionally, standard assembly metrics such as N50, the number of contigs, and BUSCO completeness (Simão et al., 2015) were also calculated to further evaluate the quality of the assembly. The N50 value was 1,559,217 bp, the total number of contigs was 53, and the BUSCO completeness score was 99.1% complete (3513 genes), with only 0.8% missing (31 genes) (using the fungi_odb9 database with BUSCO v3.0.2). The raw sequencing data were deposited in the NCBI Sequence Read Archive under accession number PRJNA1297971.

### 2.3 Gene prediction and functional annotation

Protein-coding gene prediction was performed using Diamond (v0.9.10.111). The predicted protein sequences were compared against multiple databases, including NR, eggNOG, KEGG, Swiss-Prot, GO, P450, and TCDB. The threshold for sequence alignment was set at 1e-6. GO annotation was performed using InterPro (v66.0). KEGG Orthology (KO) and pathway annotation were carried out using the KEGG KAAS (v2.1) automated annotation system. Annotated KO terms were subsequently mapped to KEGG pathways. KOG annotation was performed using eggNOG-mapper with the eggNOG (v4.5) database. In addition, RNA secondary structure prediction was performed to improve the functional annotation of non-coding RNAs (Wang et al., 2025), following recent methodological advances that integrate deep learning with structural context analysis.

### 2.4 Untargeted metabolomics

#### 2.4.1 Metabolites extraction

One milliliter of PDB culture supernatant of *P. kloeckeri* was freeze-dried. The dried sample was reconstituted in 100 μL of 80% aqueous methanol. Samples were incubated on ice for 5 min and then were centrifuged at 15,000 rpm, 4 °C for 5 min. Some of supernatant was diluted to final concentration containing 53% methanol by LC-MS grade water. The samples were subsequently transferred to a fresh Eppendorf tube and then were centrifuged at 15,000 g, 4 °C for 15 min. Finally, the supernatant was injected into the LC-MS/MS system analysis. For QC samples, equal volume of samples were taken from each experimental sample and mix them as QC samples. For blank samples, 53% methanol aqueous solution instead of experimental samples, and the pretreatment process was the same as that of experimental samples.

#### 2.4.2 UHPLC-MS/MS analysis

UHPLC–MS/MS non-targeted metabolomics of *P. kloeckeri* was performed using a Thermo Vanquish UHPLC system coupled with an Orbitrap Q Exactive HF (Biozeron Co., Shanghai). Samples were separated on a Hypersil Gold C18 column (100 × 2.1 mm, 1.9 μm) at 0.2 mL/min using a 17 min linear gradient (positive mode: 0.1% FA/H_2_O–MeOH; negative mode: 5 mM ammonium acetate buffer pH 9–MeOH). The mass spectrometer operated in both polarities with a spray voltage of 3.2 kV, capillary temperature of 320 °C, and standard gas settings. Raw data were processed in Compound Discoverer 3.1 for peak alignment, picking, and quantitation (RT tolerance 0.2 min; mass tolerance 5 ppm; S/N ≥3; minimum intensity 100,000), followed by total spectral intensity normalization. Metabolite annotation was achieved by matching MS^2^ data to mzCloud, mzVault, and MassList databases.

### 2.5 Apparent experiment

#### 2.5.1 Soybean hydroponic experiment

**Hydroponic system**. Wash *Penicillium koeckeri* spores with sterile water to prepare spore suspension. Using sterilized 1/2MS medium and adding NaCl, the final concentration of NaCl in the hydroponic solution was 80 mmol/L, simulating salt stress; Using a 650 mL transparent plastic bucket as the hydroponic device, containing 550 mL of culture medium.

**Soybean seed germination**. Disinfect the surface of soybean seeds with 10% hydrogen peroxide (soak for 20 min), rinse several times with deionized water, and place the soybean seeds on moist filter paper and sprout in a constant temperature incubator at 25 °C. Wait for the sprouts to reach about 3 cm before transplanting. Soak the spore suspension for 1 h before transplanting in the bacterial treatment group.

**Experimental design**. Three treatments were set up: CK (normal condition with 1/2 MS medium), YCK (salt stress control with 1/2 MS medium containing 80 mmol/L NaCl), and YJKS (inoculation of *P. kloeckeri* under salt stress). The NaCl concentration (80 mmol/L) was determined based on preliminary experiments, which showed that this concentration effectively inhibited soybean seed germination without causing complete seedling failure, thereby providing a reliable salt stress condition for the study. Each treatment included 5 biological replicates (5 sets of hydroponic systems and 15 soybean seeds per pot.) Samples were collected after 20 d of hydroponic cultivation. Measure plant biomass and perform root scanning using Epson Scan2.

#### 2.5.2 Experiment on improving saline alkali soil with *P. kloeckeri*

**Materials**. Soda saline alkali soil was collected from a typical disposal area in China (115°30′-115°5′E, 36°55′-37°10′N) and air-dried and sieved through a 200-mesh sieve. *P. kloeckeri* was cultivated according to the previously mentioned.

**Experimental design and physicochemical properties**. The experimental groups were set up as follows: (i) CK: Soda saline alkali soil only; (ii) JKS: Soil inoculated with *P. kloeckeri*, with a spore concentration of 10^7^ spores per gram of soil. The spore concentration was selected based on preliminary experiments, which indicated that this concentration effectively promoted soybean seed germination. Each treatment was performed with 5 biological replicates, and the experiments were repeated over a 2 m period. Additionally, the spore suspension of *P. kloeckeri* was replenished every 15 d to ensure consistent microbial inoculation.

Physicochemical Properties. pH were conducted by a pH meter (REX PHS-3E, China) in deionized water (ratio of water/solid = 5:1); Soil organic matter was measured using potassium dichromate external heating method; The spectrophotometric method is employed for the rapid determination of phosphorus (Storie, 1957); Classification and statistics of soil macroaggregates using wet screening method (Six et al., 2000).

### 2.6 Data analysis

Metabolites were annotated against the KEGG, HMDB, and LIPID Maps databases. Multivariate analyses, including PCA and PLS-DA, were performed using metaX, and univariate *t-tests* were applied to assess statistical significance. Differential metabolites were defined as those with VIP >1, *P* < 0.05, and |fold change| ≥2. Volcano plots and hierarchical clustering heatmaps (Pheatmap, R) were used to visualize differential metabolites. Pearson correlation analysis (corr and corrplot, R) was conducted, with *P* < 0.05 considered significant. Pathway enrichment analysis of differential metabolites was performed using KEGG, with pathways deemed significantly enriched at *P* < 0.05. One-way ANOVA was performed using IBM Statistical Product and Service Solutions (SPSS) Statistics for Windows (version 13), followed by *post hoc* Tukey's HSD test to assess the separation between sample means. *P* < 0.05 was considered significant. Some data visualizations were analyzed using Origin 2024 and Graphpad Prism 9.5.

### 2.7 Limitations

Methodological Constraints: Potential limitations were associated with the use of only two sequencing technologies (Illumina and PacBio), which might have introduced biases in the genomic assembly process. In addition, the resolution of untargeted metabolomics could have been influenced by the quality and complexity of the analyzed samples.

Scope of Findings: The applicability of the findings was limited to the specific strain of *P. kloeckeri* and the experimental conditions employed, and might not have fully represented the diversity of fungal species or the outcomes under different environmental conditions. Moreover, as the study focused on a single stressor (salt stress), interactions with other environmental factors could have provided further insights into the underlying microbial mechanisms.

## 3 Results

### 3.1 Genome features of *P. kloeckeri*

**Figure 1:**
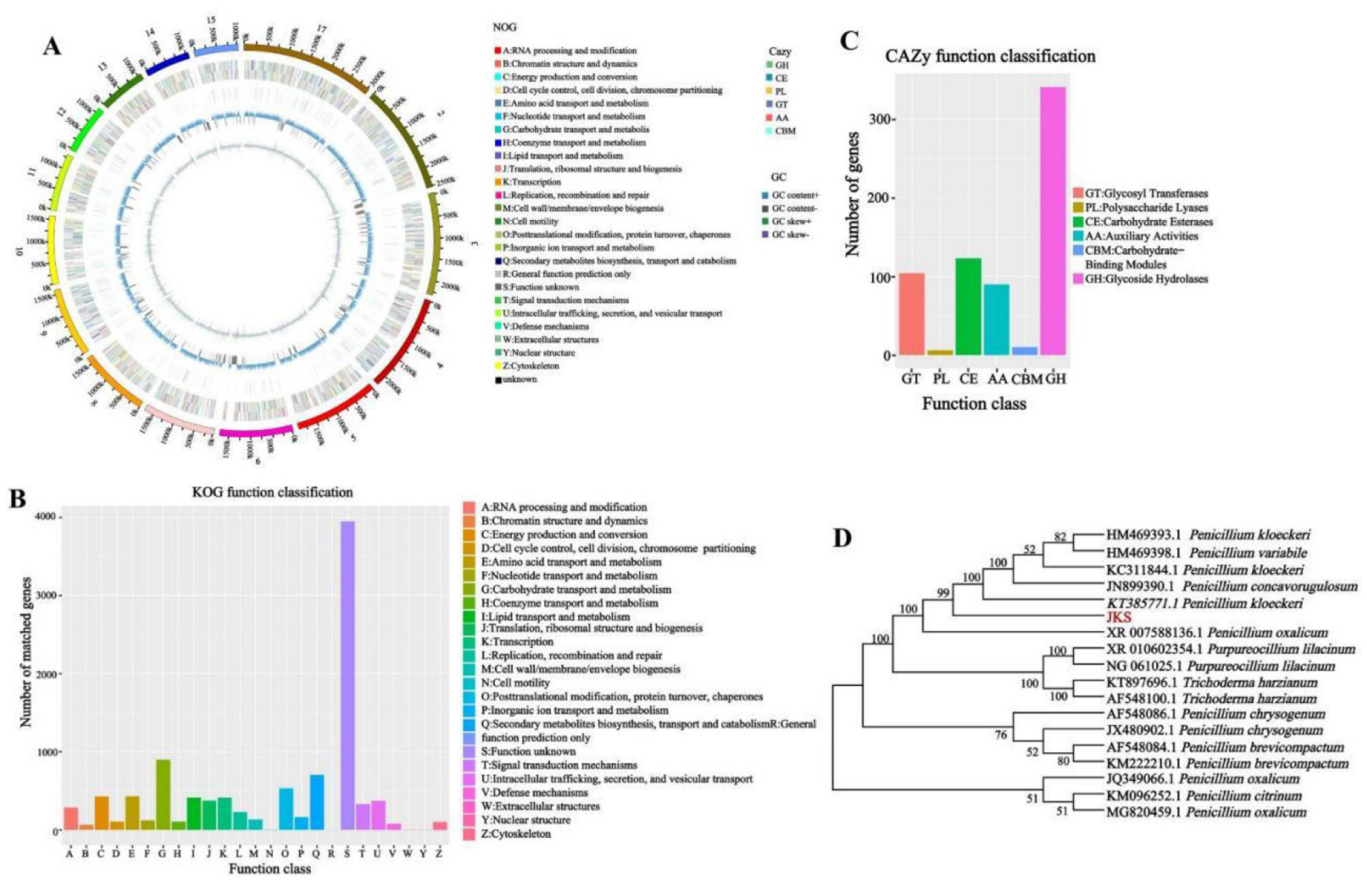
Genomic information, functional annotation, and phylogenetic tree of *P. kloeckeri*. **(A)** Whole genome circle diagram: From inside out: First lap: GC sketch (the GC content); Second circle: G + C content; Third circle: Carbohydrate-active enzymes (CAZy); Circle 4: Clusters of Orthologous Groups (COG) to which each coding DNA sequence (CDS) on the negative strand belongs; Circle 5: COG to which each CDS on the positive strand belongs; Circle 6: Scale.; **(B)** Clusters of KOG (eukaryotic orthologous groups) annotation; **(C)** Gene count distributions of carbohydrate-active enzyme (CAZy) families; **(D)** Phylogenetic tree.

The total number of genomic bases in *P. kloeckeri* is 4,392,696,002 bp, and the original total number of reads is 29,090,702. The genome size is 34.28 Mb with 41.87% GC content, a minimum sequence length of 55 bp, and a maximum sequence length of 287,510 bp. There are a total of 11,123 protein-coding genes in the genome of *P. kloeckeri*, with a total gene sequence length of 17,387,264 bp and an average length of 1,563.1 bp per gene. The total number of exons is 31,880, with an average of 2.8 exons per gene and a total exon length of 15,992,151 bp. The proportion of total exon length to genome length is 42.32%, with an average exon length of 501.6 bp and an average intron length of 67.2 bp. The total length of CDS is 15,992,151 bp, and the proportion of CDS to the genome is 42.32%. The average length of CDS is 1,437.7 bp (Figure 1A).

The eggNOG classification annotation shows that a total of 10,253 proteins obtain COG functional annotation, accounting for 92.18% of all genes. Excluding unknown functional genes, the highest number of genes involved in carbohydrate transport and metabolism is 898, accounting for 8.1%. There are 533 genes related to posttranslational modification, protein turnover, and chaperones, accounting for 4.79%. Additionally, there are 432 genes involved in amino acid transport and metabolism, accounting for 3.9%. Energy production and conversion includes 428 genes, accounting for 3.8% (Figure 1B).

The CAZy database includes carbohydrate-related enzyme families that catalyze carbohydrate degradation, modification, and biosynthesis. The CAZymes analysis reveals that glycoside hydrolases (GHs) are the major family, with 341 genes, followed by carbohydrate esterases (CEs) with 123 genes, and glycosyl transferases (GTs) with 104 genes (Figure 1C).

Phylogenetic analysis confirms the evolutionary position of the strain within *Penicillium* by comparing the obtained 18S rDNA sequences with those of related species, resulting in the construction of a phylogenetic tree (Figure 1D). The sequence alignment reveals that the strain with the highest homology is KT385771.1, corresponding to *P. kloeckeri*, with a sequence identity of 99.21%. The strain *P. kloeckeri* used in this study has been deposited at the China General Microbiological Culture Collection Center (CGMCC) under accession number CGMCC No. 41049.

### 3.2 The foundation of *P. kloeckeri* in agricultural applications

#### 3.2.1 Molecular basis of plant growth promotion and stress resistance in *P. kloeckeri*

In the genome of *P. kloeckeri*, six key genes related to IAA synthesis have been identified, including *amiE, TRP1, trpBED*, and *YUCCA*. In addition, the key enzyme acetaldehyde dehydrogenase (NAD+), which catalyzes IAA synthesis using tryptophan as a precursor, was also detected. Genes associated with zeaxanthin metabolism, such as *trnA, CKX*, and *TRIT1*, were likewise identified (Table 1).

**Table 1:** Genes associated with plant growth promotion traits and carried by *P. kloeckeri*.

**Gene Name**	**KO ID**	**Gene description**
amiE	K01426	Amidase [EC:3.5.1.4]
TRP1	K13501	Anthranilate synthase/indole-3-glycerol phosphate synthase/phosphoribosylanthranilate isomerase
trpE	K01657	Anthranilate synthase component I
trpD	K00766	Anthranilate phosphoribosyltransferase
trpB	K01696	Tryptophan synthase beta chain
YUCCA	K11816	Indole-3-pyruvate monooxygenase
trnA	K00791	Dimethylallyltransferase
CKX	K00279	Cytokinin dehydrogenase
TRIT1	K00791	tRNA dimethylallyltransferase
ALDH	K00128	Aldehyde dehydrogenase (NAD+)
entF	K02364	Enterobactin synthetase component F
mbtA	K04787	Mycobactin salicyl-AMP ligase

In the genome of *P. kloeckeri*, 18 genes associated with terpenoid biosynthesis were identified. These genes, including *GGPS1, SQLE, FDP*S, *PCYOX*1, *MV*K, *MV*D, *RCE1, FDFT1*, and *NUS1*, are primarily involved in the regulation of the terpenoid backbone biosynthesis pathway (Table 2).

**Table 2:** Genes associated with enhancing plant stress resistance carried by *P. kloeckeri*.

**Gene Name**	**KO ID**	**Gene description**
GGPS1	K00804	Type III geranylgeranyl diphosphate synthase
SQLE	K00511	Squalene monooxygenase
FDPS	K00787	Farnesyl diphosphate synthase
PCYOX1	K05906	Prenylcysteine oxidase/farnesylcysteine lyase
MVK	K00869	Mevalonate kinase
MVD	K01597	Diphosphomevalonate decarboxylase
RCE1	K08658	Prenyl protein peptidase
FDFT1	K00801	Farnesyl-diphosphate farnesyltransferase
NUS1	K19177	Dehydrodolichyl diphosphate syntase complex subunit NUS1
idi	K01823	Isopentenyl-diphosphate Delta-isomerase
STE24	K06013	Endopeptidase
ICMT	K00587	Protein-S-isoprenylcysteine O-methyltransferase
mvaK2	K00938	Phosphomevalonate kinase
DHDDS	K11778	Ditrans,polycis-polyprenyl diphosphate synthase
hexPS	K05355	Hexaprenyl-diphosphate synthase
FNTA	K05955	Protein farnesyltransferase/geranylgeranyltransferase type-1 subunit alpha
HMGCR	K00021	Hydroxymethylglutaryl-CoA reductase (NADPH)
DHDDS	K11778	Ditrans,polycis-polyprenyl diphosphate synthase

#### 3.2.2 Molecular basis for improving saline alkali soil with *P. kloeckeri*

KEGG-based metabolic pathway analysis revealed that the genome of *P. kloeckeri* contains 32 genes related to organic acid biosynthesis. These genes are involved in glycolysis (EMP), the Tricarboxylic Acid (TCA) cycle, the pentose phosphate pathway, and oxidative phosphorylation. In addition, genes encoding alkaline phosphatases, including *phoA* and *phoD*, were identified (Table 3).

**Table 3:** Genes associated with Soda saline alkali soil improvement carried by *P. kloeckeri*.

**Gene Name**	**KO ID**	**Gene description**
acnA	K01681	Aconitate hydratase
fumc	K01679	Class II fumarate hydratase,
MDH2	K00026	Malate dehydrogenase
gltA	K01647	Citrate synthase
icd	K00031	Isocitrate dehydrogenase
pckA	K01610	Phosphoenolpyruvate carboxykinase (ATP)
pdhA	K00161	Pyruvate dehydrogenase E1 component alpha subunit
phdB	K00162	Pyruvate dehydrogenase E1 component beta subunit
pdhC	K00627	Pyruvate dehydrogenase E2 component (dihydrolipoamide acetyltransferase)
aceF	K00627	Pyruvate dehydrogenase E2 component (dihydrolipoamide acetyltransferase)
pyc	K01958	Pyruvate carboxylase
SDHA	K00234	Succinate dehydrogenase (ubiquinone) flavoprotein subunit
SDHB	K00235	Succinate dehydrogenase (ubiquinone) iron-sulfur subunit
SDHC	K00236	Succinate dehydrogenase (ubiquinone) cytochrome b560 subunit
sucA	K00164	2-oxoglutarate dehydrogenase E1 component
sucB	K00658	2-oxoglutarate dehydrogenase E2 component (dihydrolipoamide succinyltransferase)
pgk	K00927	phosphoglycerate kinase
gpmI	K15633	2,3-bisphosphoglycerate-independent phosphoglycerate mutase
GPI	K01810	Glucose-6-phosphate isomerase
GAPDH	K00134	Glyceraldehyde 3-phosphate dehydrogenase
TPI	K01803	Triosephosphate isomerase (TIM)
acsA	K01907	Acetoacetyl-CoA synthetase
pgm	K01835	Phosphoglucomutase
fbaA	K01624	Class II fructose-bisphosphate aldolase
pfkA	K00850	6-phosphofructokinase 1
pyk	K00873	Pyruvate kinase
PRPS	K00948	Ribose-phosphate pyrophosphokinase
rpiB	K01808	5-phosphate isomerase B
zwf	K00036	Glucose-6-phosphate 1-dehydrogenase
deoC	K01619	Deoxyribose-phosphate aldolase
phoA	K01077	Alkaline phosphatase
phoD	K01113	Alkaline phosphatase D

#### 3.2.3 Metabolites of *P. kloeckeri*

**Figure 2:**
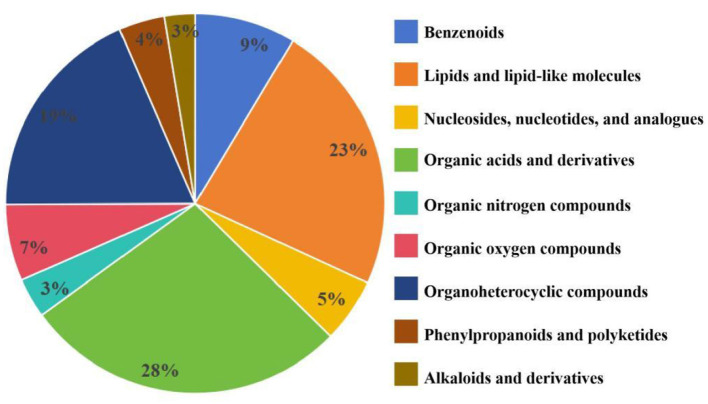
Classification of metabolites of *P. kloeckeri*.

Through identification of the metabolites produced by *P. kloeckeri*, it is found that the main metabolites include benzene ring compounds, lipids and lipid molecules, nucleosides, nucleotides, and analogs, organic acids and their derivatives, organic nitrogen compounds, organic oxygen compounds, organic heterocyclic compounds, phenylpropanoids, polyketones, and alkaloids and their derivatives. Among these, organic acids and their derivatives comprise the most substances (Figure 2). Metabolite analysis of *P. kloeckeri* revealed that it produces a variety of compounds that promote plant growth and stress resistance, including daidzein, L-phenylalanine, L-tryptophan, and L-valine (Table 4).

**Table 4:** Metabolites related to growth promotion and stress resistance produced by *P. kloeckeri*.

**Metabolite Name**	**KEGG ID**	**KEGG pathway**
Daidzein	C10208	Biosynthesis of secondary metabolites
trans-Cinnamic acid	C00423	Phenylalanine metabolism
L-Phenylalanine	C00079	Phenylalanine, tyrosine and tryptophan biosynthesis
5-Methoxyindoleacetic acid	C05660	Tryptophan metabolism
L-Tryptophan	C00078	Phenylalanine, tyrosine and tryptophan biosynthesis
Succinic semialdehyde	C00232	Alanine, aspartate and glutamate metabolism
Citric acid	C00158	Citrate cycle (TCA cycle)
L-Valine	C00183	Valine, leucine and isoleucine biosynthesis
L-Aspartic acid	C00049	Arginine biosynthesis
4-Guanidinobutyric acid	C01035	Arginine and proline metabolism
L-Pyroglutamic acid	C01879	Glutathione metabolism

### 3.3 Verification test

#### 3.3.1 The effect of *P. kloeckeri* on soybean growth promotion under salt alkali stress

**Figure 3:**
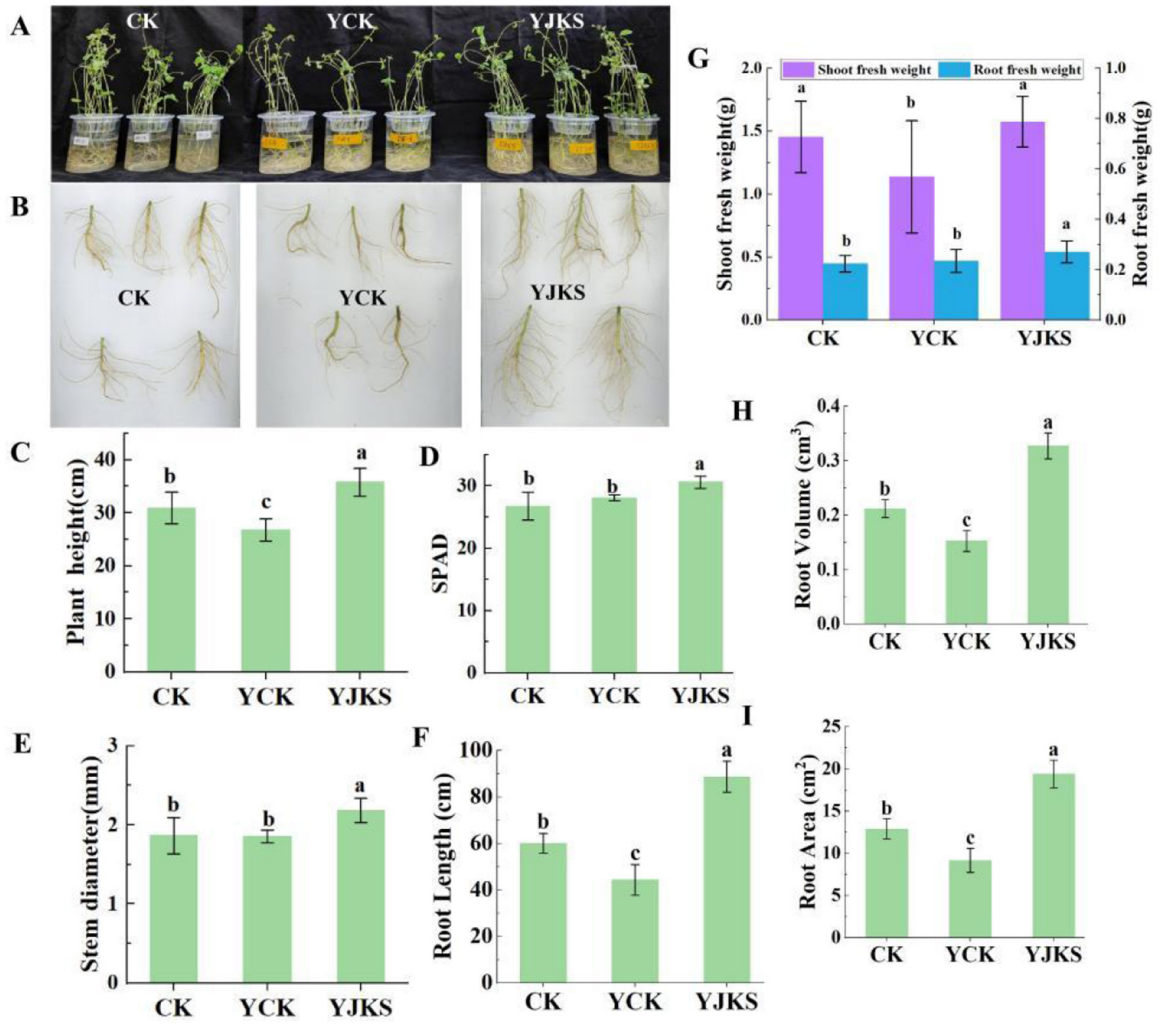
The growth status of soybean plants under different treatments **(A);** Overall growth situation; **(B)**; Soybean root, Plant height **(C)**; Chlorophyll content of soybean plants **(D);** Soybean plant stem diameter **(E)**; Root Length **(F)**; Soybean shoot and root biomass **(G);** Root Volume **(H);** Root Area **(I)** One-way ANOVA test was conducted to test the significance of difference in habitat niche breadth.

Compared with normal cultivation conditions, salt stress significantly inhibited plant growth. Under salt stress, plant height, stem diameter, shoot and root fresh weight, and chlorophyll content decreased by 13.27%, 0.54%, 21.78%, and 0.61%, respectively. Under salt stress, plants inoculated with *P. kloeckeri* showed increases in plant height (33.31%), stem diameter (17.79%), shoot fresh weight (38.39%), underground fresh weight (14.97%), and chlorophyll content (14.31%) compared with the non-inoculated control (Figures 3A–E, G). Root growth was also inhibited by salt stress, with root length, root area, and root volume reduced by 16.37%, 21.73%, and 26.68%, respectively. However, inoculation with *P. kloeckeri* markedly mitigated this inhibition. After inoculation, root length, root area, and root volume increased by 99.95%, 111.96%, and 114.80%, respectively (Figures 3F, H, I).

#### 3.3.2 The effect of *P. kloeckeri* on saline alkali soil

In the validation experiment on saline alkali soil improvement, inoculation with *P. kloeckeri* significantly reduced soil pH by 1.83% compared with the control (CK). It also increased the proportion of soil macroaggregates and microaggregates by 11.33% and 52.44%, respectively. In addition, soil organic matter content increased by 7.43%, and available phosphorus content increased by 30.17%. (Table 5).

**Table 5:** The effect of inoculation with *P. kloeckeri* on the physicochemical properties of soda saline alkali soil.

**Treatment**	**pH**	**Organic matter content(g/kg)**	**Large aggregate ratio(%)**	**Micro aggregate ratio(%)**	**Soil available phosphorus(mg)**
CK	8.21 ± 0.07a	37.66 ± 0.89b	63.45 ± 2.38b	9.84 ± 0.87b	52.16 ± 3.89b
JKS	8.06 ± 0.09b	40.46 ± 1.77a	70.64 ± 6.04a	15.00 ± 3.50a	67.90 ± 3.76a

Different lowercase letters indicate treatments that are significantly different (p < 0.05).

## 4 Discussion

Recent studies increasingly emphasize the potential of Plant Rhizosphere Growth-Promoting Microbes (PGPMs), with many efforts focused on identifying and screening effective strains (Gamalero and Glick, 2020; Samet et al., 2022). These microbes have already found broad applications in agriculture. The mechanisms by which PGPMs act can generally be divided into direct and indirect effects. Direct mechanisms involve metabolite secretion and nutrient mobilization (e.g., auxins, siderophores, phosphorus solubilization, potassium mobilization, nitrogen fixation). Indirect mechanisms improve the root microenvironment, such as alleviating saline alkaline stress or suppressing pathogens (Liu et al., 2025). Unlike the majority of microbial agents in China, which are dominated by bacteria such as *Bacillus* and *Pseudomonas*, our focus on *P. kloeckeri* highlights the potential of fungal PGPMs as alternative or complementary bioinoculants. Our genome analysis indicates that *P. kloeckeri* likely integrates both mechanisms.

### 4.1 Growth-promoting factors

Auxin plays a central role in root development and overall plant growth. Previous studies show that microbial IAA enhances root elongation and lateral root formation, facilitating nutrient uptake (Bunsupa et al., 2017; Pereira et al., 2021). In the genome of *P. kloeckeri*, we identified six genes related to Indole-3-Acetic Acid (IAA) synthesis, including *amiE, TRP1, trpBED*, and *YUCC*A. These genes encode enzymes that use tryptophan as a precursor, such as aldehyde dehydrogenase and zeaxanthin-related enzymes (trnA, CKX, TRIT1). Then, amino acids further contribute to growth promotion. For example, tryptophan is directly linked to auxin synthesis (Yao et al., 2025), glycine promotes photosynthesis in peach trees by regulating sucrose enzyme metabolic activity (Li et al., 2021). Genomic and metabolite analysis of *P. kloeckeri* showed that the strain had genes for synthesizing these amino acids, suggesting that it may use a similar mechanism to promote plant growth. Under mild salt stress, *Penicillium citrinum* was able to promote chlorophyll synthesis and plant growth, which is similar to the results of Galeano et al. (2023) on *Penicillium chrysogemun* promoting corn under salt stress.

### 4.2 Stress resistance

The genome of *P. kloeckeri* (34.28 Mb) is several times larger than those of *Bacillus subtilis* (4.0 Mb) and *Pseudomonas species* (6.5 Mb) (Li et al., 2020). A larger genome suggests a broader repertoire of genes that may regulate stress responses and plant-microbe interactions. Abiotic stress tolerance is another critical function of PGPMs. In *P. kloeckeri*, eighteen genes involved in terpenoid biosynthesis were identified. Terpenoids are widely recognized as defense-related metabolites that modulate stress signaling. In addition, amino acids such as proline and lysine also play protective roles. Proline accumulation stabilizes proteins and membranes under salt or drought stress, while lysine participates in nitrogen assimilation and chlorophyll synthesis. The presence of these metabolic pathways in *P. kloeckeri* indicates that it may directly enhance plant stress resistance, consistent with findings in other PGPMs like *Trichoderma harzianum* (Catalá et al., 2014). Functional annotation revealed that genes related to secondary metabolite biosynthesis form the second largest category in *P. kloeckeri*. This feature contrasts with many bacterial PGPMs and may explain its strong metabolic flexibility. Secondary metabolites such as EPS, VOCs, and compatible solutes (proline, trehalose, glycine betaine) are well documented to modulate genes including SOS1, HKT1, antioxidant proteins, and ethylene biosynthesis genes (Hassani et al., 2018; Kim et al., 2021). Such mechanisms enhance plant tolerance to abiotic stress and are consistent with our findings. In addition to secondary metabolites, *P. kloeckeri* possesses extensive carbohydrate-active enzyme (CAZyme) genes, including glycoside hydrolases (341 genes), carbohydrate esterases (123 genes), and glycosyltransferases (104 genes). It provides support for the degradation and utilization of complex carbohydrates of *P. kloeckeri* to obtain more energy and carbon sources (Starke et al., 2021; Zhang et al., 2025) and can also help *Penicillium kermitense* to occupy ecological advantages in the competitive ecological environment.

### 4.3 Soil improvement potential

Soil nutrient availability and pH balance are key determinants of crop productivity in saline alkaline conditions. *P. kloeckeri* harbors thirty two genes related to organic acid synthesis, spanning multiple metabolic pathways such as glycolysis, the TCA cycle, the pentose phosphate pathway, and oxidative phosphorylation. Importantly, we identified *phoA* and *phoD*, which encode alkaline phosphatases. These enzymes are known to release inorganic phosphate from organic compounds, thereby improving phosphorus availability in soil (Liu et al., 2025). By secreting organic acids, *P. kloeckeri* can also reduce soil pH, a mechanism previously described in *Penicillium oxalicum* (Jiang et al., 2020). *Penicillium bairei* is recognized for its strong phosphorus-solubilizing capabilities (Brazhnikova et al., 2022). In addition, *Penicillium oxalicum* increased micro aggregate stability in acidic soils, enhanced phosphorus dissolution, and reduced lead bioavailability (Tong et al., 2024). Thus, the combination of acid production and phosphatase activity positions *P. kloeckeri* as a promising agent for saline alkaline soil improvement.

## 5 Conclusion

In this study, whole genome sequencing and comparative genomic analysis of *P. kloeckeri* were performed. Functional genes associated with auxin (IAA) synthesis, daidzein production, siderophore secretion, and organic acid metabolism were identified. These findings emphasize the potential role of *P. kloeckeri* in promoting plant growth and improving soil health. Metabolite profiling confirmed the production of daidzein and multiple amino acids, which supports the genomic predictions. Pot experiments further validated the beneficial effects of *P. kloecker*i. Under salt stress, *P. kloeckeri* inoculation significantly enhanced soybean performance, with stem diameter increasing by 17.79%, and root length, area, and volume improving by nearly 100-115% compared to the uninoculated control. Additionally, inoculation improved saline alkaline soil conditions by reducing pH, enhancing soil aggregate stability, increasing organic matter content, and boosting available phosphorus levels. These findings position *P. kloeckeri* as a promising fungal inoculant with dual benefits for plant growth and soil health, particularly under stress conditions. Future research should focus on elucidating the molecular mechanisms that mediate these effects, conducting field-scale trials to validate its robustness in diverse environments, and developing strategies for its large-scale commercial application in sustainable agriculture.

## Data Availability

The datasets presented in this study are publicly available. This data can be found here: https://www.ncbi.nlm.nih.gov/sra, accession number PRJNA1297971.
