# Supplementary material for: Whole genome sequence of Penicillium kloeckeri and insight into its growth-promoting, saline alkaline tolerance properties
Source: Front Microbiol. 2025 Oct 10;16:1675497. doi: 10.3389/fmicb.2025.1675497 (PMC12550956; doi:10.3389/fmicb.2025.1675497)
Supplement: Supplementary file 1 [file Supplementary_file_1.docx]

**Supplementary information**

Whole genome sequence of *Penicillium kloeckeri* and insight into its growth-promoting, saline-alkaline tolerance properties

**Names of authors:** Wenxiao Cui ^a,b,c^, Yafen Wu^a,b,c^, Bin Ni^c^，Jia Cao^a,b,c*^

**Present addresses of authors:**

a. College of Resources and Environmental Sciences, China Agricultural University, Beijing 100193, P. R. China

b. Beijing Key Laboratory of Biodiversity and Organic Farming, China Agricultural University, Beijing 100193, P. R. China

c. State Key Laboratory of Nutrient Use and Management, College of Resources and Environmental Sciences, China Agricultural University, 100193 Beijing, China

***Corresponding author:**

Jia Cao, E-mail: [jia.cao@cau.edu.cn](mailto:jia.cao@cau.edu.cn); Tel.: +86 18610168832

**
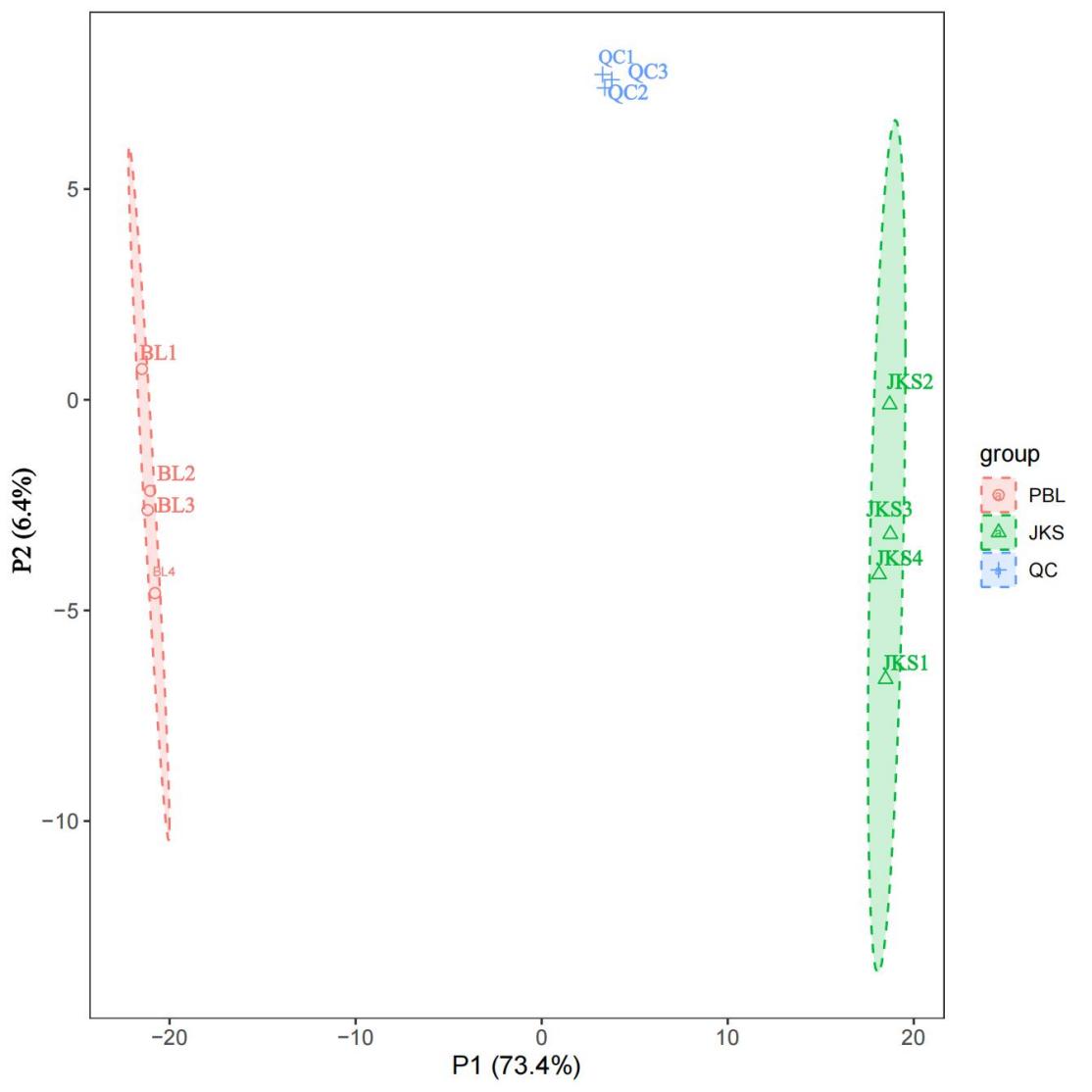
**

**Figure S1.** PCA plots of QC samples and metabolic group samples
